# Single* versus* Double Skin Preparation for Infection Prevention in Proximal Humeral Fracture Surgery

**DOI:** 10.1155/2018/8509527

**Published:** 2018-10-14

**Authors:** Davide Blonna, Valeria Allizond, Enrico Bellato, Giuliana Banche, Anna Maria Cuffini, Filippo Castoldi, Roberto Rossi

**Affiliations:** ^1^Surgical Sciences Department, Mauriziano-Umberto I Hospital, University of Torino, 10128 Turin, Italy; ^2^Public Health and Pediatrics Department, University of Torino, 10126 Turin, Italy; ^3^Surgical Sciences Department, San Luigi Gonzaga Hospital, University of Torino, 10043 Orbassano (Turin), Italy

## Abstract

Preoperative skin preparation plays a major role in preventing postoperative infections. This study aims to compare a single skin preparation (povidone iodine) with a double skin preparation (chlorhexidine gluconate followed by povidone iodine). Forty patients affected by proximal humeral fracture were included in the study. The day of surgery the two skin preparation strategies were performed in the same shoulder, divided into two areas, at the level of the deltopectoral approach. Skin swabs were collected from each area and subjected to microbiological analysis. Both skin preparations significantly reduced the positive culture rate. Coagulase-negative staphylococci (CoNS) dropped from 92.5% to 40% and to 7.5% after the single and double skin preparation (p<0.001), respectively. The positivity rate was reduced from 50% to 17.5% (p=0.002) and from 27.5% to 0% (p= 0.001) for* Propionibacterium acnes* and* Staphylococcus aureus,* respectively, with no difference between the two preparations. The double skin preparation had a more significant effect on bacterial load against CoNS compared to the single skin preparation (p<0.001* versus* p= 0.015). In conclusion, both the approaches reduced* S. aureus *and* P. acnes* skin load, whereas the double skin preparation is more effective than the single one against CoNS. In light of our findings, preoperative strategies able to reduce bacterial load could potentially increase the final efficacy of perioperative traditional skin preparations.

## 1. Introduction

Despite the fact that many types of progress have been made in the field of shoulder surgery over the last decade, postoperative infection remains an orthopaedic surgeon threat since it is a major source of both patient morbidity and rising health care costs [1, 2].

In shoulder surgery the most common infective bacteria are coagulase-negative* Staphylococcus* species (CoNS) and* Propionibacterium acnes*, which are part of the normal skin flora in the healthy adult population [3–7]. Considering their commensal role in the skin, preoperative skin preparation is considered to play a role in preventing postoperative infections. Several studies have proved different skin preparation strategy efficacy on the positive culture rate [8–12]. Saltzman* et al. *[12] compared three different skin preparation options to eradicate bacteria from the shoulder region. Both chlorhexidine gluconate and iodophor, in addition to isopropyl alcohol, were more effective than povidone iodine alone in eliminating CoNS; but the 7-15% of the shoulders still had positive cultures for* P. acnes, *with no significant difference in terms of the efficacy in eliminating this bacterium from the shoulder region by each preparation. The reason why* P. acnes* is resistant to common skin preparation solutions may be related to its presence primarily in the dermal layer [4, 5]; hence alternative strategies need to be tested to prevent such complications [8–14]. Literature regarding* P. acnes* load, with respect to positive culture rate, is still lacking probably because its measurement is a more expensive and time-consuming procedure, even if it provides important insights on the real effect of common skin preparations. It is conceivable that different skin preparations can have similar effects on the positive culture rate, but different outcomes in terms of bacterial load.

We previously showed that a double skin preparation (chlorhexidine gluconate followed by painting with iodopovidone iodine/alcohol) can protect against acute deep infections, in the surgical treatment for proximal humeral fractures (PHF), with respect to single skin preparation with iodopovidone iodine and alcohol [15]. The aim of our present study is to test the efficacy of these two strategies in reducing the* P. acnes, *CoNS, and* Staphylococcus aureus* load in the skin of the deltopectoral approach.

## 2. Materials and Methods

### 2.1. Patients

In light of the Italian law, no institutional review board approval was mandatory for this study. The study has been performed in accordance with the ethical standards in the 1964 Declaration of Helsinki and has been carried out in accordance with relevant regulations of the Italian National Health Care System. An informed consent was obtained for all patients.

Forty patients affected by displaced PHF and scheduled for reduction and fixation were included in the study during the period September 2015-January 2017. Of the 40 patients, 32 were females. The average age was 66 ± 9.3 year old (45-88 years). All the patients were recruited in the emergency room the day of the trauma after they accepted to undergo surgical treatment. All patients had their affected shoulder placed in a bandage for pain control and were discharged until the day of surgery.

### 2.2. Sample Collection and Skin Preparation

The day of surgery, after the bandage removal in the operative room, the first skin culture swab, used as control, was collected from the skin area of the deltopectoral approach, immediately before skin preparation and approximately 15 minutes after antibiotic prophylaxis (2 g of cefazolin). The culture swab was rubbed twice, for 10 cm distally to the tip of the coracoid.

**Figure 1:**
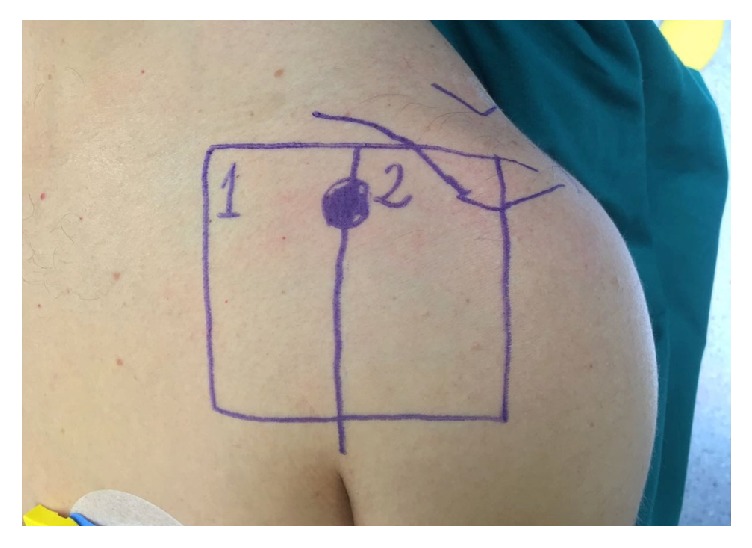
The skin area of the deltopectoral approach was divided into 2 subareas, respectively medial (#1) and lateral (#2) to the incision line.

Afterward, the area of the deltopectoral approach was longitudinally splitted into two areas, one medial and one lateral to the planned incision line (Figure 1).

The single skin preparation consisted of a painting technique using two consecutive sterile gauzes soaked with 1% povidone iodine (10% of iodine available) and 50% isopropyl alcohol (Poviderm; Nuova Farmec, Settimo di Pescantina, Verona, Italy). The skin swabs were collected 3 minutes after the application of the antiseptic solution to let it evaporate.

For the double skin preparation, the skin was first vigorously scrubbed/rubbed with a sponge and a soap solution of 4% chlorhexidine gluconate (Neoxidina Mani; Nuova Farmec). After one minute of scrubbing, the remaining solution was removed with a sterile gauze. The second part of the skin preparation consisted of a painting with 10% povidone iodine and 50% isopropyl alcohol as described for the single skin preparation.

The two skin preparation strategies were performed on the same shoulder, one in the medial and the other in the lateral area of the surgical field. A second skin culture swab was collected from each skin area (i.e., medial* versus* lateral) that was randomly chosen. Randomization was performed using the online software Research Randomizer (http://www.randomizer.org, Version 4.0-Urbaniak, G. C., & Plous, S. 2015). Hence, a total of three culture swabs were collected in every case, immediately placed into sterile culture swab tubes with Amies transport medium (Becton Dickinson Italia S.p.a., BD, Buccinasco, Milan, Italy) and brought to the Bacteriology and Mycology Laboratory of the Department of Public Health and Pediatrics, University of Torino, Turin (Italy), within one hour for microbiological analysis.

At this point, all the shoulders were prepared again with a double skin preparation. For ethical reasons, we decided to not leave part of the shoulder prepared with only a single skin, in light of the data reported in our previous multicentre study [15].

The patient follow-up was obtained at 1 week, 2 weeks, 4 weeks, 3 months, 6 months, and 1 year after surgery to detect potential infections, either superficial or deep.

### 2.3. Microbiological Assays

At the Bacteriology and Mycology Laboratory the three skin swabs were analyzed within one hour as previously described [15]. Briefly, for each sample 100 *μ*L of serial 10-fold dilutions in 3 ml of saline solution (0.9% NaCl) were prepared and spread on Nutrient Agar (NA; Oxoid S.p.A., Milan) for the colony-forming units (C.F.U.)/mL determination of aerobic bacteria, on Mannitol Salt Agar (MSA; Merck Bracco, Milan) for staphylococci and on Schaedler Agar plus 5% blood (BD) for anaerobic bacteria.

The C.F.U. number was recorded after incubation at 37°C for 24-48 hours under aerobic conditions for aerobic bacteria and for up to 7 to 14 days under strictly anaerobic conditions within an anaerobic system (Gaspak EZ anaerobe pouch system kit, BD) for obligate/facultative anaerobic bacteria; and the isolated microorganism identification was carried out by the biochemical assays API systems as indicated by manufacturers' instructions (BioMérieux, Rome, Italy). The higher number of bacterial species obtained in each media was used for different bacterial species counts. The antimicrobial susceptibility test was performed for all* S. aureus* isolates to detect potential multiresistant bacteria (CLSI document M100–S22 2012).

### 2.4. Statistical Analysis

The cohort size required to achieve 80% power at alpha = 0.05 was 39 patients. The data were based on the assumption that the double skin preparation would be associated with a positive culture rate of 7% versus 31% in the single skin preparation group. Although the measurement of bacterial load, and not the positive cultural rate, was the primary outcomes of our study, the power analysis was based on the positive cultural rate because precise information was available on the bacterial load. The comparison of the C.F.U. number between pre- and postskin preparations was performed using a paired T-test. The comparison of the positive cultural rate between pre- and postskin preparation was performed using the McNemar test for paired proportion.

The comparison between single and double skin preparations was performed using the Fisher test for proportion and a T-test for comparison between averages (unpaired comparison).

A multiple regression analysis was applied to find potential correlation between independent variables (age, gender, and delay of surgery) and dependent variables (bacterial load before skin preparation and after skin preparations). A logistic regression analysis was performed to find out potential risk factors for positive swab cultures.

## 3. Results and Discussion

### 3.1. Results

#### 3.1.1. Positive Cultures

The skin swabs were collected 3.4 ± 2.6 days (0-14) after the trauma. Before skin preparation, 92.5% (37/40) of the skin cultures were positive for CoNS (72.5% [29/40] of which were positive for* S. epidermidis*), 50% (20/40) for* P. acnes,* and 27.5% (11/40) for* S. aureus*. The rate of positive cultures for* P. acnes* before skin preparation was similar between male (4/8, 50%) and female patients (16/30, 53%).

**Figure 2:**
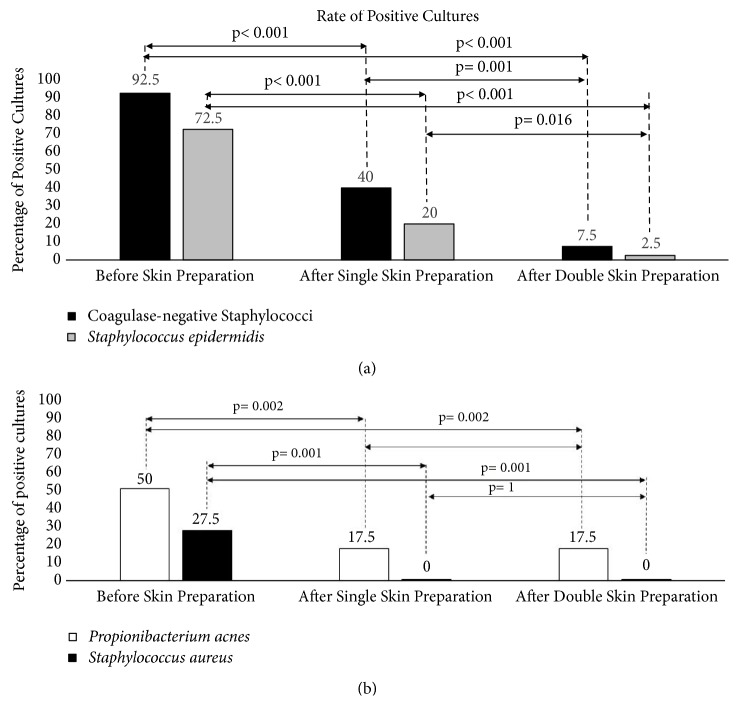
The two graphs portray the percentage of cultures before and after skin preparation (both single and double) positive for CoNS (a) and for* P. acnes* and* S. aureus* (b).

Both the skin preparations were able to significantly reduce the positive culture rate compared to the controls (Figures 2(a) and 2(b)). Of note, the double skin preparation was more effective against CoNS (7.5%* versus* 40%, p= 0.001), including* S. epidermidis *(2.5%* versus* 20%, p= 0.016), but not against* S. aureus *or* P. acnes*, for which both preparations had the same effect.

#### 3.1.2. Bacterial Load

**Figure 3:**
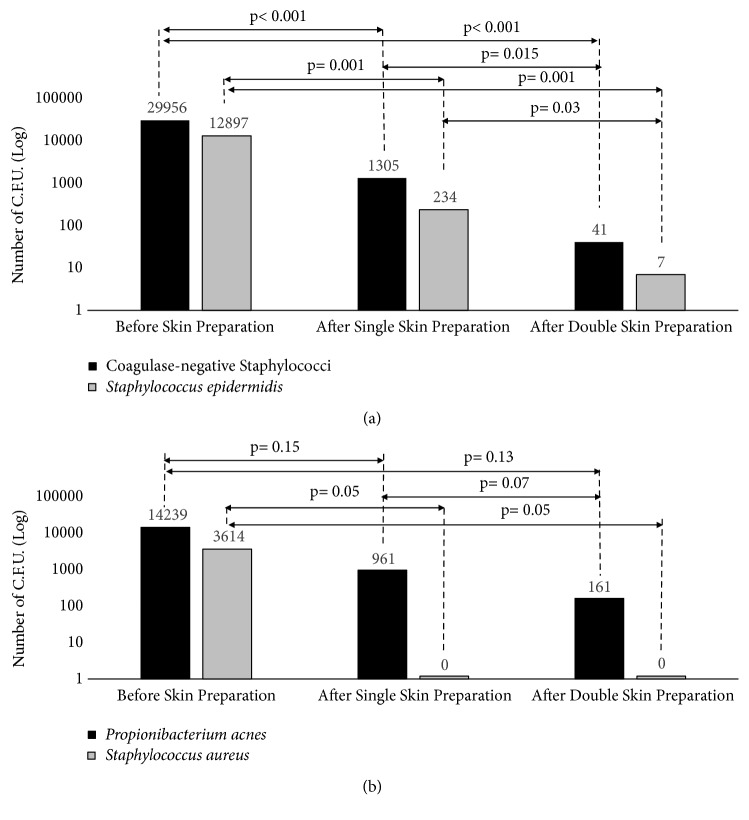
The two graphs portray the number of C.F.U. before and after skin preparation (both single and double) for CoNS (a) and for* P. acnes* and* S. aureus* (b).

The CoNS load analysis confirmed a significant effect induced by both skin preparations. The double skin preparation had a higher effect on the bacterial load against CoNS compared to the single skin preparation (p<0.001* versus* p= 0.015) (Figure 3(a)). Considering* S. aureus,* both the skin preparations were effective in reducing the bacterial count to zero (Figure 3(b)). Considering* P. acnes* load, both the skin preparations reduced its load on the skin: even if the difference between the two skin preparation strategies was not statistical significant (9.61*∗*10^2^* versus* 1.61*∗*10^2^, p= 0.07) (Figure 3(b)).

The mean* P. acnes *load was 2.73*∗*10^3^ ± 3.45*∗*10^3^ C.F.U. among the female patients and 3.00*∗*10^4^  ± 2.78*∗*10^3^ C.F.U. among the male patients (p= 0.04). In the 32 females, the mean bacterial load was reduced from 2.73*∗*10^3^  ± 2.07*∗*10^3^ C.F.U. to 4.00*∗*10^2^ ± 3.00*∗*10^2^ C.F.U. after single skin preparation (p= 0.04), and to 7.8*∗*10^1^ ± 2.48*∗*10^2^ C.F.U. after double skin preparation (p= 0.05). The comparison between single and double skin preparation within women was not significant (p=0.2). In the 8 males the mean bacterial load was 3.00*∗*10^4^ ± 1.55*∗*10^3^ C.F.U. and was reduced to 1.16*∗*10^3^ ± 1.05*∗*10^2^ after single skin preparation and to 1.50*∗*10^2^ ± 1.27*∗*10^2^ C.F.U. after double skin preparation (considering the low number of male patients, statistical analysis was not performed).

None of the cultures positive for* S. aureus*, subjected to antimicrobial assay, was found to be multiantibiotic resistant. None of the patients were affected by infection during the follow-up period.

At multiple regression analysis the variable “Female” was statistically related to the load of* P. acnes* before skin preparation (r = -0.33, p= 0.04). The logistic regression analysis revealed that the only independent variables related to the rate of positive culture was the delay of surgery that was a risk factor for* S. aureus* (ODDS = 1.55, 95%CI 1.2-2.2, p=0.01).

#### 3.1.3. *P. acnes Post Hoc* Analysis

**Figure 4:**
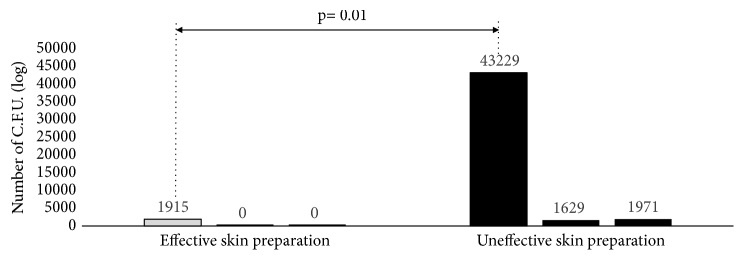
*P. acnes post hoc* analysis.

A* post hoc* analysis was run to detect potential correlation between gender and* P. acnes* and variables associated with failure of the skin preparation, defined as a positive culture after skin preparation. Patients with a failure in one of the two skin preparations were compared with patients in whom both the disinfection methods were able to eradicate* P. acnes* from the skin. Patients who experienced a failure in one of the two skin preparations had a significantly higher bacterial load before skin disinfection. Whereas the average bacteria load before skin disinfection was 4.32*∗*10^4^ ± 3.03*∗*10^3^ C.F.U. in the patients who had a failure in the skin preparations, only a mean of 1.91*∗*10^3^ ± 2.5*∗*10^2^ C.F.U. was observed in the patients in whom both skin preparation procedures eliminated* P. acnes* (Mann-Whitney test; independent samples, p = 0.01) (Figure 4).

### 3.2. Discussion

The aim of the present study was to test the effect on bacterial growth of two different preoperative skin preparations: the single (povidone iodine and alcohol) and the double (chlorhexidine gluconate, followed by povidone iodine and alcohol). Our hypothesis was that the approach consisting in scrubbing and rubbing the operative site with the double skin preparation would be more effective than the single one against the common infective bacteria of the shoulder (i.e., CoNS,* S. aureus *and* P. acnes*). Our original hypothesis was not entirely supported by the obtained results.

The double skin preparation was more effective than the single one against CoNS, both on positive bacterial culture rate and bacterial load, in eliminating potential pathogenic bacteria from the surgical field. These data support our previous findings, where the double skin preparation was a significant protective factor against acute infection after PHF surgery [15]. The rationale behind the positive effect of the double skin preparation could be the combination of the mechanical effect of the scrub with the soap solution, with the chemical effect of the chlorhexidine (4%), povidone iodine (1%) and alcohol (50%). All these preoperative solutions have proven to be effective against bacteria with different mechanism [16].

Saltzman* et al*. [12] found that ChloraPrep (2% chlorhexidine gluconate and 70% isopropyl alcohol) and DuraPrep (0.7% iodophor and 74% isopropyl alcohol) were more effective than povidone iodine scrub and paint (0.75% iodine scrub and 1.0% iodine paint) at eliminating CoNS from the shoulder. With ChloraPrep as the most effective solution, our outcomes seem to confirm these data. In fact, we found that the association among chlorhexidine gluconate, povidone iodine, and isopropyl alcohol was able to significantly reduce the bacterial load and the rate of cultures positive for CoNS; the effect was more prominent when compared to that obtained after the preparation with povidone iodine and alcohol only. These data suggest that the main effect of the double skin preparation could be either the mechanical effect of the scrubbing, the chlorhexidine gluconate bactericidal effect, or the combination of these two. The comparison between the scrub only and the painting technique has never been tested in shoulder skin preparation. In a prospective randomized trial on abdominal surgery, paint only was equivalent to scrub and paint in preoperative skin preparation [17]. However, in this study the main outcome was the wound infection rate and not the bacterial load or rate of positive cultures.

In contrast to Saltzman* et al*. [12] we found that one-third of the shoulders were positive for* S. aureus* before skin preparation, probably due to the different cohort of patients included in our study. These patients were older and affected by PHF, with subsequent impossibility to clean the area of the shoulder since the day of trauma. This could justify the increased rate of some pathogenic bacteria found in the area of the deltopectoral approach [18]. We can here speculate that both the skin preparations were equally effective against* S. aureus*, without any apparent differences, due to the low bacterial load before skin preparation. In fact, when the bacterial load is higher, such as for CoNS, the more significant effect of the double skin preparation probably becomes more relevant.

Our study showed a suboptimal effect of both the skin preparation strategies against* P. acnes*. Although the rate of positive cultures for* P. acnes* was significantly reduced after both skin solutions, a rate of 17.5% positive cultures cannot be considered a satisfactory outcome. In comparison, the rate of positive cultures for CoNS was only 7.5% after double skin preparation, with a mean bacterial load of 4.10*∗*10^1^ C.F.U. The different efficacy against bacteria is even more relevant if we consider that the CoNS load, before skin preparation, was more than twice that of* P. acnes*. These results confirm the data reported by Phadnis* et al*. [19]. Interestingly, they found that* P. acnes* was cultured in 42% of the prepreparation skin surface swabs, and in 14% of the postpreparation skin surface swabs, despite 2 g of intravenous cefazolin and skin preparation with 2% chlorhexidine gluconate and 70% isopropyl alcohol. These rates were very similar to our data, suggesting that adding a vigorous scrub and povidone iodine does not contribute to* P. acnes* elimination from the skin area of the deltopectoral approach. Unfortunately, they did not measure the bacterial load, so our studies are not fully comparable.

The present study is the first to consider the bacterial load rather than the rate of positive cultures alone to measure the effect of skin preparation against potential pathogenic bacteria of the shoulder skin. This different approach highlighted discrepant results when considering different outcomes (i.e., the C.F.U. number or the rate of positive cultures). In fact, considering only the rate of positive cultures a substantial equal effect was observed against* P. acnes* between the two methods of skin preparation. In contrast, considering the* P. acnes* load, the double skin preparation seems to be 6 times more effective than the single one: however, this outcome does not reach the statistical level of significance, probably because the study was underpowered for the bacterial load analysis.

Furthermore, after measuring the bacterial load, our study showed that the efficacy of the skin preparation against* P. acnes* is related to the bacterial load before skin preparation. In patients with a low bacterial load, both the skin preparations were effective. Conversely, in patients with higher bacterial load both the skin preparation techniques were more likely to be ineffective.

Regarding the role of patients' gender, we noticed that* P. acnes* load, before the skin preparation, was significantly higher in the male population. This could justify the greater infection rate sustained by* P. acnes* in the first group reported in the literature, but further studies are needed [20]. Our results should in fact be considered cautiously in light of the low number of men compared to women enrolled in our study.

In light of our findings, preoperative strategies able to reduce the bacterial load could potentially increase the final efficacy of perioperative traditional skin preparations. This hypothesis could justify the efficacy of topical benzoyl peroxide on* P. acnes* reduction during shoulder surgery showed by Sabetta* et al*. [11]. These authors found that a skin preparation with benzoyl peroxide cream 48 hours before surgery was able to reduce the rate of* P. acnes* positive cultures. It is conceivable that the benzoyl peroxide reduced both the rate of positive cultures and the bacterial load, increasing the subsequent effect of the 2% chlorhexidine gluconate and 70% isopropyl alcohol.

The present study has some limitations. First, the study was underpowered for the bacterial load analysis. The power analysis was in fact weighted on the rate of positive cultures and not on the bacterial load. This choice was forced by the lack of data regarding the bacterial load in the shoulder. Second, the study population was not homogeneous in terms of gender. Finally, the small number of samples here included highlights the need for further studies in larger population before definitive conclusions can be made.

## 4. Conclusions

In conclusion, in our cohort of patients affected by PHF, the double skin preparation was more effective against CoNS, compared to the single one; whereas its effect against* P. acnes* was less effective than expected, despite these results we suggest that patients affected by PHF should undergo preoperative double skin preparation. Further studies are mandatory to investigate the role of other strategies in reducing the preoperative* P. acnes* load in the surgical field.

## Data Availability

The microbiological data used to support the findings of this study are included within the article.
